# ASSOCIATION BETWEEN LONG-TERM EXPOSURE TO AIR POLLUTION AND LOWER URINARY TRACT SYMPTOMS IN KOREAN OLDER MEN

**DOI:** 10.1093/geroni/igad104.3568

**Published:** 2023-12-21

**Authors:** Youngmin Jee

**Affiliations:** Seoul National University, Seoul, Republic of Korea

## Abstract

Low Urinary Tract Symptoms(LUTS) is one of the common symptoms in older men and is important factor in terms of quality of life. The purpose of this research was to investigate the association between long-term exposure to air pollution and LUTS in korean older men. The study participants were recruited from a health checkup center of Seoul National University Hospital from January 1st, 2015 to December 31st, 2019. A total of 7,979 participants were aged over 65 years and underwent screening health checkup including International prostate symptom score (IPSS) for LUTS. To evaluate the exposure to air pollution, hourly measurements of air pollutants from nationwide air quality monitoring sites were utilized. The associations between exposure to air pollutants and LUTS were analyzed using logistic analysis in a crude model and an adjusted model, the latter of which included covariates. Most air pollutants including PM10, NO2, SO2, and CO were all significantly associated with LUTS in crude models, but the correlations were no longer significant in the adjusted models. PM2.5 exposures showed significantly positive association with voiding symptoms in adjusted model (OR, 1.07; 95% CI, 1.02~1.13; p=0.012). The associations of other air pollutants with voiding and storage symptoms showed negative tendency in unadjusted model, and some of them were significant. However, the associations were no longer significant in adjusted model. This study demonstrated that air pollutants including PM10, NO2, SO2, and CO are associated with LUTS, particularly PM2.5 showed a positive correlation with voiding symptoms of LUTS in the adjusted model.

